# Gan-Qing-Ning Formula Inhibits the Growth of Hepatocellular Carcinoma by Promoting Apoptosis and Inhibiting Angiogenesis in H_22_ Tumor-Bearing Mice

**DOI:** 10.1155/2020/6376912

**Published:** 2020-08-06

**Authors:** Fan-Yan Zeng, Kai-Li Zhao, Le-Zhen Lin, Ying Deng, Si Qin, Jin-Rong Ye, Zeng-Qiong Huang

**Affiliations:** ^1^Affiliated Tumor Hospital, Guangxi Medical University, Nanning, Guangxi 530021, China; ^2^College of Pharmacy, Guangxi Medical University, Nanning, Guangxi 530021, China

## Abstract

**Objective:**

Gang-Qing-Ning (GQN) is a traditional Chinese medicine formula that has been used in the treatment of hepatocellular carcinoma (HCC) in the folk population for decades. However, scientific validation is still necessary to lend credibility to the traditional use of GQN against HCC. This study investigates the antitumor effect of GQN on H_22_ tumor-bearing mice and its possible mechanism.

**Methods:**

Fifty H_22_ tumor-bearing mice were randomly assigned to five groups. Three groups were treated with high, medium, and low dosages of GQN (27.68, 13.84, and 6.92 g/kg, respectively); the positive control group was treated with cytoxan (CTX) (20 mg/kg) and the model group was treated with normal saline. After 10 days' treatment, the tumor inhibitory rates were calculated. Pathological changes in tumor tissue were observed, and the key proteins and genes of the mitochondrial apoptosis pathway were measured, as well as the mRNA expression levels of VEGF in tumor tissue.

**Results:**

The tumor inhibitory rates of high, medium, and low dosages of GQN groups were 47.39%, 38.26%, and 22.17%, respectively. The high dosage of the GQN group significantly increased the protein and mRNA expression levels of Bax, Cyt-C, and cleaved Caspase 3 (or Caspase 3) (*P* < 0.01) but decreased the expression levels of Bcl-2, VEGF, and microvessel density (MVD) (*P* < 0.01).

**Conclusions:**

The high dosage of GQN can significantly inhibit the tumor growth in H_22_ tumor-bearing mice. It exerts the antitumor effect by enhancing proapoptotic factors and inhibiting the antiapoptotic factor of the mitochondrial apoptosis pathway and inhibiting tumor angiogenesis.

## 1. Introduction

Hepatocellular carcinoma (HCC) is currently the 4^th^ most common cancer and the leading cause of cancer death in China [1], and the 3^rd^ leading cause of cancer death in the world [2–4]. HCC is the main type of liver cancer and accounts for 90% of primary liver cancers [5–8]. In China, liver cancer is the leading cause of cancer death in men before the age of 60 years and occurs most frequently in East China, followed by Southwest China, and Northwest China has the lowest incidence rate of liver cancer [1]. HCC has become a major disease that threats human health and life. Although great efforts have been made in the treatment of HCC, the cure rate and survival time of patients with HCC are still not optimistic.

The main methods for HCC treatment include surgical therapy, transcatheter arterial chemoembolization, radiotherapy, and targeted therapy, among others. In addition, traditional Chinese medicines play an important role in the treatment of HCC. Gan-Qing-Ning (GQN) is a traditional Chinese medicinal formula composed of 19 medicinal materials, such as *Eupolyphaga steleophage*, *Hirudo*, *Hedyotis diffusa*, and *Scutellaria barbata* (Table 1). GQN has been used in the treatment of HCC in the folk population for decades and can significantly prolong patient survival. The main medicines of *Eupolyphaga steleophage* and *Hedyotis diffusa* in GQN have been proven to inhibit tumor growth via the apoptosis pathway or inhibiting the proliferation of tumor cells [9–14]. These medicines also inhibit tumor angiogenesis [12, 15, 16]. Other medicines in the formula, such as *Scutellaria barbata* [17], *Agrimonia pilosa* [18], and *Hirudo* [19], have been found to promote apoptosis of tumor or macrophage cells via the mitochondrial apoptotic pathway.

The mitochondrial apoptotic pathway is one of the most important pathways in tumor cell apoptosis. Classical activation of this pathway results from the action of the upstream signal molecules Bax and Bak on the mitochondrial membrane and the opening of the mitochondrial permeable transition pore (MPTP), which triggers the apoptotic process and induces apoptosis by releasing apoptotic factors, for example, cytochrome C (Cyt-C), into the cytoplasm [20]. Cyt-C, apoptotic protease activating factor (Apaf-1), and procaspase-9 coactivate the downstream protein procaspase 3 and promote cell apoptosis. In this signaling pathway, proapoptotic proteins, such as Bax, Cyt-C, Caspase 3, and Caspase 9, and antiapoptotic proteins, such as Bcl-2 and Bcl-x, coregulate apoptosis. It has been confirmed that dysregulation of the mitochondrial apoptotic pathway and tumor angiogenesis play important roles in the occurrence and development of HCC [20–23].

Based on these previous studies, we hypothesized that the effect of GQN in the treatment of HCC is mediated by the promotion of apoptosis of tumor cells and inhibition of tumor angiogenesis. Therefore, this study was designed to observe the effect of GQN on the growth of HCC by establishing an H_22_ tumor-bearing mouse model and to reveal the effects of GQN on apoptosis and tumor angiogenesis by measuring the expression of the apoptosis-related proteins and genes Bax, Bcl-2, Cyt-C, and cleaved Caspase 3 (or Caspase 3) and by determining the tumor microvessel density (MVD) and expression of vascular endothelial growth factor (VEGF), which are key indicators of tumor angiogenesis [24–26].

## 2. Materials and Methods

### 2.1. Experimental Drugs

Gan-Qing-Ning (GQN) is composed of 19 medicinal materials that were separated into two portions: portion I and portion II (Table 1). All of the medicinal materials and the 0.9% sodium chloride injection produced by Chenxin Pharmaceutical Co., Ltd., were purchased from the Guangxi Laobaixing Pharmacy Co., Ltd. (Nanning, China). Cytoxan (CTX), produced by Jiangsu Shengdi Pharmaceutical Co., Ltd., with 200 mg in each ampoule was purchased from the Pharmacy of the First Affiliated Hospital of Guangxi Medical University (Nanning, China).

### 2.2. Medicinal Preparation

The medicinal materials of portion I of GQN were ground into powder, sifted through a 100-mesh sieve, and mixed well. The materials of portion II were extracted 3 times with water at 10 times the weight of the materials at 100°C for one hour each time. The extract was concentrated to 2.17 g of medicinal materials per milliliter. The fine powder of portion I and the concentrated solution of portion II were mixed, and the concentrations were adjusted to 1.384, 0.692, and 0.346 g/ml with deionized water. These suspensions were stored at 4°C for subsequent use. CTX was diluted with 0.9% sodium chloride injection to a final concentration of 2 mg/ml.

### 2.3. Main Reagents

The protease inhibitor, BCA Protein Assay Kit, and Prestained Protein Ladder were purchased from Thermo Fisher Scientific (USA). Cleaved Caspase 3, Cyt-C, Bax, Bcl-2, VEGF, and CD34 antibodies were purchased from Abcam (Shanghai) Trading Co., Ltd. (Shanghai, China). The GAPDH antibody was purchased from Cell Signaling Technology Inc. (USA). IRDye800CW Goat anti-Rabbit IgG (H + L) was purchased from LI-COR Bioscience (USA). The Fluorescent Quantitative PCR Kit, First Strand cDNA Synthesis Kit, and RNA Extraction Kit were purchased from Takara Bio, Inc. (Japan). The RNA primers were designed and synthesized by Takara Bio, Inc. (Japan). The DAB Substrate Kit (DA1010) was purchased from Beijing Solarbio Science & Technology Co., Ltd. (Beijing). Peroxidase Conjugated Goat anti-Rabbit IgG (H + L) (ZB-2301) was purchased from Beijing Zhongshan Jinqiao Biotechnology Co., Ltd. (Beijing).

### 2.4. Main Instruments

The following instruments were used in this work: UW620H electronic scales (Shimazu production institute, Japan), a 5810R high speed refrigerated centrifuge (Eppendorf China Limited), a MicroCL17R refrigerated centrifuge and Nanodrop3000 nucleic acid protein analyzer (Thermo Fisher Scientific, USA), a CKX-41 inverted microscope (Olympus, Japan), a 7500 fluorescence quantitative PCR and 9700 PCR instrument amplification system (Applied Biosystems, Inc., USA), an odyssey infrared fluorescence scanning imaging system (LI-COR Bioscience, USA), SpectraMaxPlus384 ELIASA (Hong Kong molecular instrument co. ltd, China), and DMR + Q550 Pathological image analyzer (LEICA Co., Germany).

### 2.5. Animals and Cells

Male Kunming mice (20 ± 2 g) were purchased from the Laboratory Animal Center of Guangxi Medical University, China [License No. SCXK (Gui) 2014-0002]. Mice were maintained in a specific pathogen-free (SPF) environment. Murine H_22_ hepatocellular carcinoma cells were provided by the Department of Pharmacology of Guangxi Medical University. Before inoculation in mice, the H_22_ hepatocellular carcinoma cells were diluted to a concentration of 1 × 10^7^ cells/ml with normal saline. An amount of 0.2 ml of the cell suspension was inoculated in the abdominal cavity of mice, and ascites were passaged 3 times. All animal studies were approved and strictly performed according to the protocols issued by the Animal Care & Welfare Committee of Guangxi Medical University.

### 2.6. Animal Modeling, Grouping, and Drug Regimen

Eleven days after the injection of H_22_ hepatocellular carcinoma cells into third-generation mice, milk-white ascites of mice were extracted under aseptic conditions and adjusted to a concentration of 1 × 10^7^ cells/ml with normal saline. An amount of 0.2 ml (2 × 10^6^) of hepatocellular carcinoma cells was subcutaneously inoculated into the right axilla of each mouse to establish the hepatocellular carcinoma model.

After inoculation for 24 h, 50 mice were randomly divided into 5 groups. The positive control group received an intraperitoneal injection of CTX at a dosage of 20 mg/kg once every other day for 10 days. The model group received an intragastric administration of normal saline. The high-, medium- and low-dose GQN groups (GQN (H), (M), and (L), respectively) received an intragastric administration of GQN suspension at dosages of 27.68, 13.84, and 6.92 g/kg, respectively. The model group and the three GQN groups were treated twice daily with 20 ml/kg for 10 days. The mice were weighed every two days.

### 2.7. Tumor Growth Inhibitory Rate, Organ Index, and Histology

Twenty-four hours after the last administration, mice were sacrificed by cervical dislocation, and the tumors, thymuses, and spleens were isolated and weighed immediately. The tumor growth inhibitory rate (IR) and organ index were calculated by the following formulas:

Tumor growth inhibitory rate (%) = (average tumor weight of model group − average tumor weight of treated group)/average tumor weight of model group × 100%.

Organ index = average organ weight (milligram)/average body weight (gram).

Tumor tissue was fixed in a 10% neutral formaldehyde solution for 48 h and embedded in paraffin. Paraffin sections (4 *μ*m thick) were cut, certain sections were stained with hematoxylin and eosin (H&E) for histologic observation, and other sections were used for immunohistochemistry detection.

### 2.8. Immunohistochemistry Detection of Tumor Tissue

Paraffin sections were stained with immunohistochemistry methods for determination of MVD and VEGF. These determinations were performed using primary antibodies against CD34 (1 : 100 dilution), which is a vascular marker factor, and VEGF (1 : 1000 dilution). All sections were counterstained with hematoxylin and observed by light microscopy (×400).

Positive expression of the CD34, which appears as diffuse cytoplasmic brown-yellow or brown particles, was used to determine MVD in tumor tissue. The MVD value of each section was counted by choosing the three areas with the densest microvessels under light microscopy (×400). The mean values of MVD were used in statistical analyses.

Positive expression of VEGF presents as a brown-yellow color in the cytoplasm or cytomembrane. The proportion and staining intensity of positive cells were observed by an image analyzer and were assigned scores as follows. Positive proportions of 1–25%, 26–50%, 51–75%, and 76–100% were assigned scores of 1, 2, 3, and 4, respectively. The weak, medium, and strong staining intensities of positive cells were assigned scores of 1, 2, and 3, respectively. The VEGF expression level was counted by adding the score of the positive proportion to the score of the staining intensity. The final scores were used in statistical analyses.

### 2.9. Western Blotting Analysis of the Protein Expression of Bax, Bcl-2, Cyt-C, and Cleaved Caspase 3 in Tumor Tissue

Tumor tissues were ground into powder in liquid nitrogen, transferred into tissue lysis buffer, and centrifuged to obtained tissue proteins (supernatant). The protein concentration was measured by the BCA protein assay kit. Equal quantities of protein (60 *μ*g) were separated by sodium dodecyl sulfate polyacrylamide gel electrophoresis (SDS-PAGE) with a 5% concentration gel and 12% separation gel and were subsequently transferred to nitrocellulose membranes. The membranes were stained with ponceau, rinsed with ultrapure water, blocked with skim milk in Tris-buffered saline containing 0.05% Tween-20 (TBST) for 1 h, and rinsed 3 times with TBST. The membranes were incubated with primary antibodies against Bax (1 : 2000), Bcl-2 (1 : 2000),Cyt-C (1 : 5000), and cleaved Caspase 3 (1 : 2000) overnight at 4°C. After 3 rinses with TBST, the membranes were incubated with a fluorescence labeled secondary antibody for 90 min and then rinsed 3 times with TBST. The glyceraldehyde-3-phosphate dehydrogenase (GAPDH) gene served as an internal reference. Finally, the membranes were scanned by the Odyssey Bicolor infrared laser imaging system, and the optical densities were analyzed with Odyssey and Image J 1.8.0 software.

### 2.10. Real-Time Fluorescence Quantitative PCR (RT-PCR) Detection of the mRNA Expression of Bax, Bcl-2, Caspase 3, and VEGF in Tumor Tissue

Extraction of total RNAs from tumor tissue was performed by following the manufacturer's instructions for the RNA extraction kit. cDNA was synthesized from 0.5 *μ*g of total RNAs by reverse transcription using first strand cDNA synthesis kit. RT-PCR was performed on a 7500 fluorescence quantitative PCR amplification system (Applied Biosystems, USA). The amplification conditions were set as follows: stage 1 at 95°C for 30 s, 40 cycles of stage 2 at 95°C for 5 s, and 60°C for 34 s. The primers are shown in Table 2.

The Ct value was used to refer to the cycle threshold, and the ΔCt value is the Ct value of target genes after subtracting the Ct value of GAPDH. ΔΔCt was calculated by subtracting the Ct values of the model group from the Ct value of the different groups. Finally, the 2^−ΔΔCt^ calculation method was used to calculate the mRNA expression level of target genes related to the GAPDH gene.

### 2.11. Statistical Analysis

All data are presented as the mean ± SD, and one-way ANOVA was used to compare significant differences between groups via SPSS 19.0 software. *P* < 0.05 was considered statistically significant.

## 3. Results

### 3.1. Effects of GQN on Tumor Growth, the Thymus and Spleen Indices, and Tumor Histology

**Figure 1:**
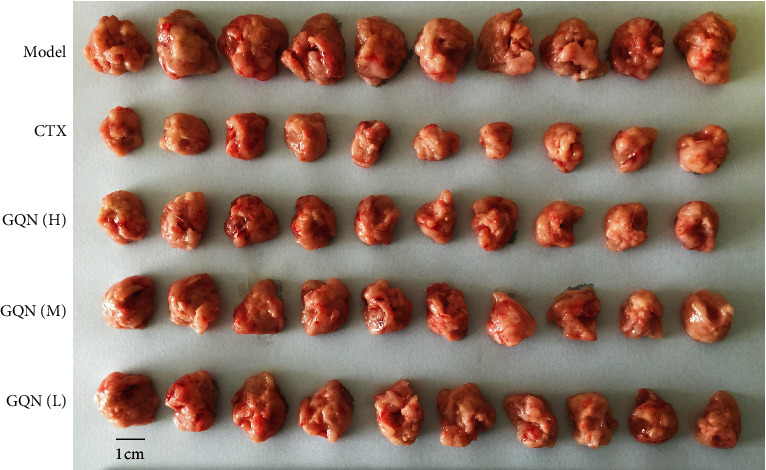
Optical photo of tumors from different treated groups.

To understand the effect of GQN on tumor growth, we weighed tumors from each group at the end of study. As shown in Figure 1 and Table 3, the tumor weight of the treatment groups significantly decreased (*P* < 0.01) compared with that of the model group. The CTX group showed the largest decrease in tumor weight and presented a significant difference compared with those of the GQN groups (*P* < 0.01). The tumor weight of the GQN groups decreased with increasing dosage, and, correspondingly, the inhibitory rate gradually increased. It should be noted that the inhibitory rate of the GQN (H) group (47.39%) was more than twice that of the GQN (L) group but was 10 percentage points lower than that of the CTX group. Obviously, the CTX group had the most significant inhibitory effect on tumor growth, followed by the GQN (H) group, and the dosage of GQN had a significant effect on tumor growth (Figure 1).

It is known that the immune system has a significant effect on tumor growth. To evaluate the effects of GQN on the immune system, we calculated the thymus and spleen indices of all mice. The results (Table 4) showed that the thymus and spleen indices of the GQN groups were not significantly different compared with those of the model group. In the CTX group, the thymus index remarkably decreased (*P* < 0.01) but showed no significant difference in the spleen index compared with that of the model group. Compared with the CTX group, the GQN groups showed significant differences of both the thymus and spleen indices (*P* < 0.05 or *P* < 0.01). Our results indicate that the effect of GQN on immune system function is limited.

**Figure 2:**
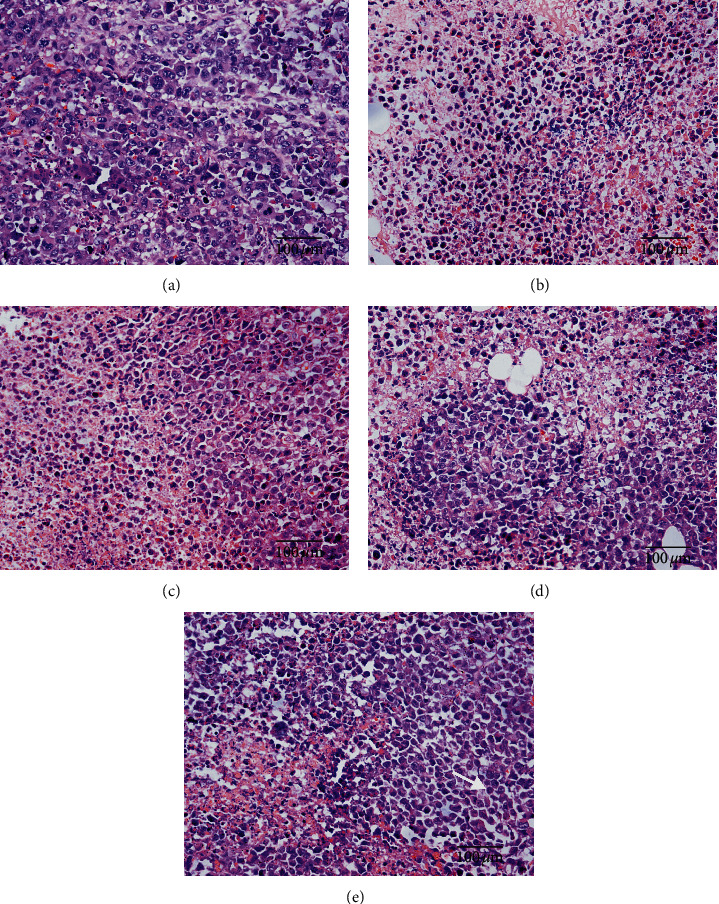
Effects of GQN on the histopathology of tumor tissue in H_22_ tumor-bearing mice. Sections were stained with hematoxylin and eosin (H&E) and were observed at a magnification of ×400. (a) Model group, (b) CTX, (c) GQN (H), (d) GQN (M), and (e) GQN (L).

H&E staining of the model group exhibited dark purple and double or multinucleated cells with vague margins and different morphologies and sizes (Figure 2(a)). Cells from the CTX and GQN (H) groups, which had clear boundaries, were smaller than those from the model group, and their cell densities remarkably decreased compared with those of the model group (Figures 2(b) and 2(c)). Although the changes in cell morphology and density in the GQN (M) and GQN (L) groups were not as obvious as those in the GQN (H) group, they showed inhibitory effects on the growth of tumor cells, and GQN (M) was more effective than GQN (L) (Figures 2(d) and 2(e)).

### 3.2. Effect of GQN on the Protein Levels of Bax, Bcl-2, Cyt-C, and Cleaved Caspase 3 in Tumor Tissue

**Figure 3:**
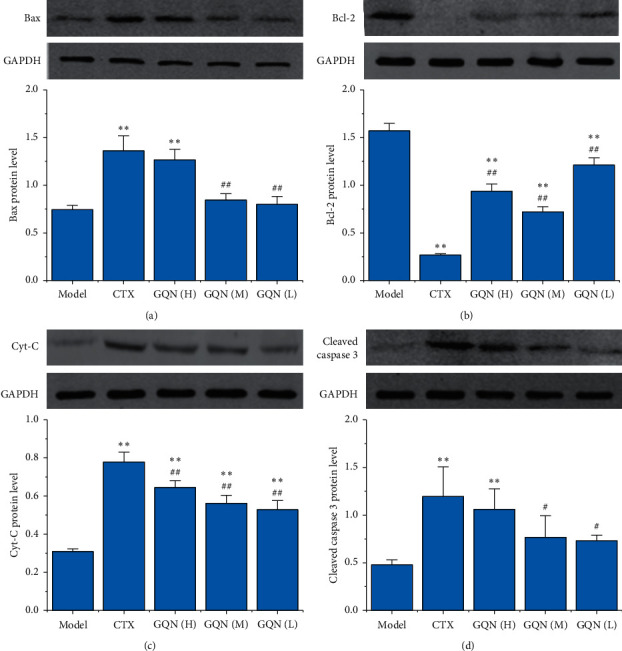
Effects of GQN on the protein expression levels of Bax, Bcl-2, Cyt-C, and cleaved caspase 3 in tumor tissue from H_22_ tumor-bearing mice. ^*∗*^*P* < 0.05 and ^*∗∗*^*P* < 0.01 compared with the model group. ^#^*P* < 0.05 and ^##^*P* < 0.01 compared with the CTX group.

To evaluate the effect of GQN on proteins associated with the mitochondrial apoptotic pathway, we detected the protein levels of Bax, Bcl-2 Cyt-C, and cleaved Caspase 3 at the end of the treatment.  Figure 3(a) shows that the Bax levels significantly increased only in the CTX and GQN (H) groups and that the levels in these two groups were almost identical. However, the level of Bcl-2 significantly decreased in all treatment groups, and the GQN groups displayed significant differences compared with the CTX group (Figure 3(b)). The level of Cyt-C significantly increased in all treatment groups, and the difference was significant among the CTX and GQN groups (Figure 3(c)). However, level of cleaved Caspase 3 significantly increased only in the CTX and GQN (H) groups, and no significant difference was observed between the CTX and GQN (H) groups (Figure 3(d)).

### 3.3. Effect of GQN on the mRNA Expression of Bax, Bcl-2, Caspase 3, and VEGF in Tumor Tissue

**Figure 4:**
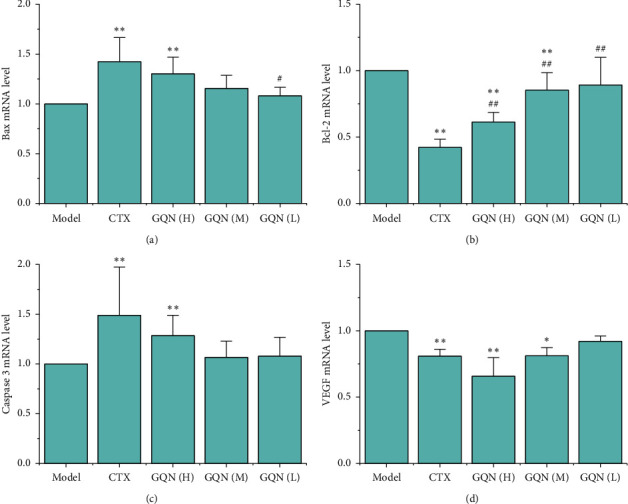
Effects of GQN on the mRNA expression of Bax, Bcl-2, Caspase 3, and VEGF in tumor tissue from H_22_ tumor-bearing mice. ^*∗*^*P* < 0.05 and ^*∗∗*^*P* < 0.01 compared with the model group. ^#^*P* < 0.05 and ^##^*P* < 0.01 compared with the CTX group.

To elucidate the possible mechanism of GQN in the antitumor effect, we measured the gene transcription of Bax, Bcl-2, and Caspase 3, which are associated with apoptosis, and VEGF, a signaling factor for angiogenesis in tumor tissues. As shown in Figure 4(a), the CTX and GQN (H) treatments significantly increased the mRNA expression of Bax, but no significant difference was noted between the two groups. However, the mRNA expression of Bcl-2 was significantly decreased in the treatment groups, except GQN (L), and was significantly different between the CTX and GQN groups (Figure 4(b)). For Caspase 3, only CTX and GQN (H) showed significantly increased mRNA expression, and no significant difference was observed between them (Figure 4(c)). Similar to Bcl-2, the mRNA expression of VEGF was significantly decreased in the CTX, GQN (H) and GQN (M) groups compared with that in the model group (*P* < 0.01 and *P* < 0.05), and the GQN (H) led to more decreased expression of VEGF than CTX, although no significant difference was noted between these groups (Figure 4(d)).

### 3.4. Effect of GQN on Tumor Angiogenesis in Tumor Tissue

**Figure 5:**
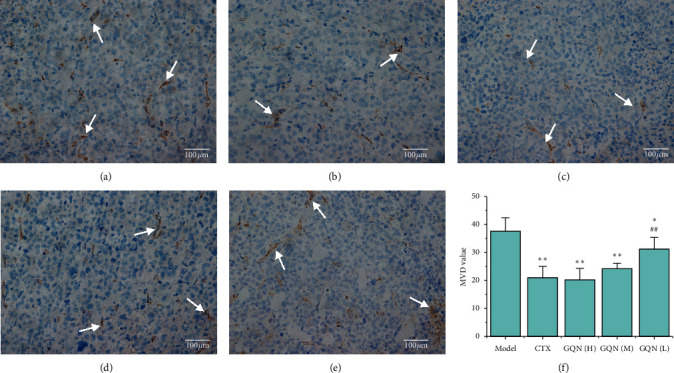
Effects of GQN on the MVD level in tumor tissue from H_22_ tumor-bearing mice. Figures 5(a)–5(e): immunohistochemistry observation (×400), white arrows indicate the positive staining. (a) Model group, (b) CTX, (c) GQN (H), (d) GQN (M), (e) GQN (L), and (f) MVD value. ^*∗*^*P* < 0.05 and ^*∗∗*^*P* < 0.01 compared with the model group. ^#^*P* < 0.05 and ^##^*P* < 0.01 compared with the CTX group.

The inhibition of tumor angiogenesis is an important mechanism in the antitumor effect. To evaluate the effect of GQN on tumor angiogenesis, we detected MVD and VEGF in tumor tissues by performing immunohistochemistry experiments. MVD is the number of microvessels observed per unit area. Here, we obtained the MVD values by measuring the positive expression of CD34, because CD34 is a vascular marker factor expressed mainly in neovascular endothelial cells of tumor. Its positive expression can be considered as tumor angiogenesis. The MVD experimental results showed a brown-yellow cytoplasm with brown particles spread in selected cells from each group (Figures 5(a)to 5(e)), and the staining intensity of GQN (H) was the lowest (Figure 5(c)). Furthermore, all the treatment groups showed significantly reduced MVD in tumor tissues (*P* < 0.01 and *P* < 0.05), and GQN (H) and GQN (M) showed no significant difference compared with CTX (Figure 5(f)). These results indicate that the treatment groups can inhibit the formation of tumor microvessels.

**Figure 6:**
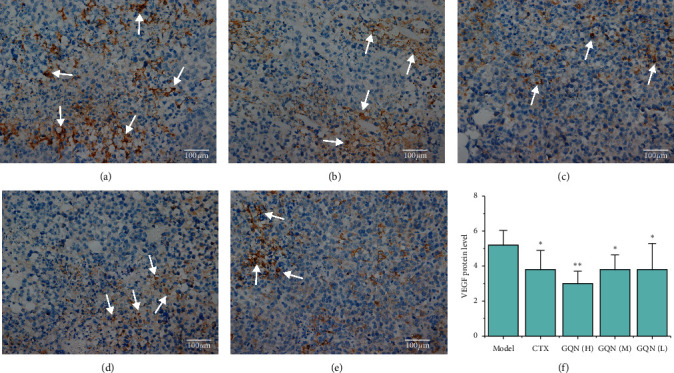
Effects of GQN on the protein expression of VEGF in tumor tissue from H_22_ tumor-bearing mice. Figures 6(a)–6(e): immunohistochemistry observation (×400), white arrows indicate the positive staining: (a) model group, (b) CTX, (c) GQN (H), (d) GQN (M), (e) GQN (L), and (f) VEGF protein level. ^*∗*^*P* < 0.05 and ^*∗∗*^*P* < 0.01 compared with the model group. ^#^*P* < 0.05 and ^##^*P* < 0.01 compared with the CTX group.

The VEGF experimental results showed that the cytoplasm and cytomembrane of certain cells in each group were stained brown-yellow in color (Figures 6(a)to 6(e)). Moreover, all the treatment groups had significantly decreased expression of VEGF (*P* < 0.05 and *P* < 0.01), and the expression of VEGF decreased with the increasing dosage of GQN (Figure 6(f)). It is worth noting that GQN (H) achieved the best inhibitory effect on VEGF expression but showed no significant difference compared with CTX (Figure 6(f)).

## 4. Discussion

It is known that many traditional Chinese medicines inhibit tumor growth by affecting the immune system. The most important organs of the immune system are the thymus and spleen, and they participate in cellular and humoral immunity, which can be a primary defense against cancer [27, 28]. The main function of thymus is to produce T lymphocyte and thymosin and low thymus index means low immunity. While the spleen is rich in macrophages and lymphocytes, and it can produce immunoglobulin, the increase of spleen index indicates the enhancement of immunity. Thus, the thymus and spleen indices are often used to evaluate immune function, and boosting immunity is helpful for tumor therapy. Our results showed that there was no significant effect of GQN on the thymus and spleen indices. But it should be noted that the spleen indices of GQN were increased compared with model group, which indicates that GQN has a certain effect on improving immunity, but the effect is not significant. These results indicate that the antitumor effect of GQN might be achieved mainly by other means.

“Like cures like” is an important principle of traditional Chinese medicine in the treatment of tumors. Based on this theory, the medicinal materials of GQN, such as *Eupolyphaga steleophage* and *Hirudo*, could be used to kill tumor cells because they are believed to be slightly poisonous in Chinese medicine. Previous studies have proven that these two medicinal materials are effective in promoting apoptosis in tumor cells [10, 11, 19]. Therefore, in this paper, the effect of GQN on the mitochondrial apoptotic pathway was determined by documenting the expression of the proapoptotic proteins Bax, Cyt-C, and cleaved Caspase 3 and the antiapoptotic protein Bcl-2 in tumor tissue to explain its antihepatocellular carcinoma effect.

In the mitochondrial apoptotic pathway, the upstream signal molecule Bax is activated by apoptosis stimulating factors, which leads to the release of Cyt-C. Cyt-C promotes the formation of an apoptotic complex, and Caspase 3 is cleaved into two subunits. As a result, Caspase 3 is activated and induces apoptosis [20, 23]. Our results showed that GQN (H) significantly increased the expression of the Bax, Cyt-C, and cleaved Caspase 3 proteins as well as the Bax and Caspase 3 genes and also significantly decreased the expression of Bcl-2 (Figures 2 and 3). Although the effects of the GQN (M) and GQN (L) groups were not as significant as that of GQN (H), they showed a trend of increasing the expression of proapoptotic proteins and genes while decreasing the expression of antiapoptotic proteins and genes. These results indicate that GQN inhibits the growth and development of HCC by regulating the mitochondrial apoptosis pathway.

As mentioned above, the main medicines in GQN, such as *Eupolyphaga steleophage* [10, 11] and *Hedyotis diffusa* [13, 29], as well as other medicines, such as *Scutellaria barbata* [17], *Agrimonia pilosa* [30], and *Hirudo* [19], exhibit activity in promoting apoptosis of tumor cells by affecting the mitochondrial apoptotic pathway. Although we do not know exactly which chemical components in GQN play key roles in promoting apoptosis, selected components, such as hirudin of Hirudo [31], quercetin and methylanthraquinone of *Hedyotis diffusa* [14, 32], scutebarbatine of *Scutellaria barbata* [33], and agrimoniin of *Agrimonia pilosa* [34], have been confirmed to have antitumor effects. Furthermore, the ethanol extract of *Eupolyphaga steleophage*, which mainly contains fatty acids [11], total coumarins of *Hedyotis diffusa* [35] and polysaccharide isolated from *Agrimonia pilosa* [18], has been reported to promote H_22_ tumor cell, myelodysplastic syndrome SMK-1 cell, and human osteosarcoma U-2 OS cell apoptosis by increasing proapoptotic proteins and/or decreasing antiapoptotic proteins, respectively. The regulation of the mitochondrial apoptotic pathway by GQN should involve the contribution, to a large extent, of the mentioned components because medicinal materials that contain these components are viewed as the main medicines in the formula. However, the chemical compositions of traditional Chinese medicine formulas are quite complicated, and they usually have multitarget and multipath efficacy. Therefore, GQN probably inhibits the growth and development of HCC by affecting multiple signaling pathways.

HCC is considered to be a hypervascular tumor, and tumor growth, invasion, and metastasis are closely related to angiogenesis [36]. Of the angiogenic factors, VEGF is the most common in tumor tissue and is of prognostic value in HCC [37, 38]. The VEGF expression level is positively correlated with tumor MVD [24–26]. Our results showed that the protein and gene expression of VEGF as well as MVD were significantly inhibited by GQN and CTX, and, moreover, the inhibitory effect of the GQN (H) group was superior to that of the other treatment groups (Figures 3(d), 4, and 5). These results are consistent with the tumor growth inhibitory rate and suggest that the antitumor effect of GQN is mediated by inhibiting tumor angiogenesis.

The antitumor angiogenesis of GQN should primarily be attributed to the main medicinal materials of *Eupolyphaga steleophage*, *Hedyotis diffusa*, *Hirudo,* and *Scutellaria barbata* in the formula because chemical components from these medicines, such as fibrinolytic protein [39], 4-vinylphenol [40], hirudin [41], and polysaccharide [42], can significantly inhibit tumor growth by downregulating the expression of VEGF or have significant antiangiogenesis effects. These researches supported our inference that one of the mechanisms of GQN in antihepatocellular carcinoma is the inhibition of tumor angiogenesis. However, we still did not know exactly which components of GQN play an antitumor role. Also we have not yet determined the content of the main chemical components or the major antitumor components of GQN. In subsequent studies, the high performance liquid chromatography (HPLC) should be used to control the quality of GQN.

In addition to regulating the mitochondrial apoptosis pathway and inhibiting tumor angiogenesis, GQN probably affects other signaling pathways. For instance, the ethanol extract of *Eupolyphaga steleophage* exhibits an antitumor effect by regulating the MAPK and KDR signaling pathways [9, 15], *Hedyotis diffusa* has been demonstrated to have an anticolorectal cancer effect via regulation of the TGF*β* and IL-6/STAT 3 signaling pathways [43, 44], and Radix curcumae, which contains diterpenoid C [45], has an antitumor effect by regulating the MAPK signaling pathway. Therefore, other mechanisms of GQN should exist in antihepatocellular carcinoma. Unfortunately, the in vitro antitumor experiments have not been carried out in this paper, and the effects of GQN on other signaling pathways remain unknown. For further studies, serum pharmacology method can be used in in vitro experiments (suggested by reviewer) to confirm the antihepatocarcinoma effect of GQN and reveal its effect on other signaling pathways. And a toxicological study of GQN should also be conducted.

## 5. Conclusions

In summary, we confirmed the antihepatocarcinoma effect of the traditional Chinese medicine formula GQN, which is used in the treatment of hepatocarcinoma in the folk population. GQN, especially the high-dosage group, exhibited a significant inhibitory effect on tumor growth. The antitumor effect of GQN is mediated its ability to regulate of the mitochondrial apoptosis pathway and inhibit tumor angiogenesis, that is, increasing the proapoptotic factors Bax, Cyt-C, and Caspase 3 and decreasing the antiapoptotic factor Bcl-2, as well as downregulating the angiogenic factor VEGF and decreasing MVD. Our results suggest that GQN is an effective formula for the treatment of HCC.

## Figures and Tables

**Table 1:** Contents of Gan-Qing-Ning (GQN) formula.

Portion	Chinese name	Botanical name	Common name	Weight (g)	Part used
I	Tu Bie Chong	*Eupolyphaga sinensis* walker	Eupolyphaga steleophage	30	Whole
	San Qi	*Panax notoginseng* (Burk.) F. H. Chen	Radix et rhizoma notoginseng	30	Root and rhizome
	Shui Zhi	*Hirudo nipponica* Whitman	Hirudo	20	Whole
	Ji Nei Jin	*Callus gallus domesticus* Brisson	Endothelium corneum	30	Gizzard lining
	Yu Jin	*Curcuma longa* L.	Radix curcumae	30	Root tuber
	Huang Qi	*Astragalus membranaceus* (Fisch.) Bunge	Radix astragali	30	Root
	Fu Ling	*Poria cocos* (Schw.)wolf	Poria	30	Sclerotium
	Dang Shen	*Codonopsis pilosula* (Franch.) Nannf.	Radix codonopsis	30	Root

II	Bai Hua She She Cao	*Hedyotis diffusa* Willd	Hedyotis diffusa	35	Herba
	Ban Zhi Lian	*Scutellaria barbata* D. Don	Scutellaria barbata	35	Herba
	Xian He Cao	*Agrimonia pilosa* Ledeb.	Agrimonia pilosa	35	Flower, fruid, leaf and stem
	Cu Ye Rong	*Ficus hirta* Vahl.	Radix fici simplicissimae	50	Root
	Bai Zhu	*Atractylodes macrocephala* Koidz.	Rhizoma atractylodis	30	Rhizome
	Cang Zhu	*Atractylodes lancea* (Thunb.) DC.	Rhizoma atractylodis	30	Rhizome
	Lu Lu Tong	*Liquidambar formosana* Hance	Fructus liquidambaris	20	Infructescence
	Zhi Zi	*Gardenia jasminoides* Ellis	Fructus gardenia	15	Fruid
	Da Fu Pi	*Areca catechu* L.	Pericarpium arecae	15	Pericarp
	Niu Xi	*Achyranthes bidentata* Blume	Radix achyranthis Bidentatae	15	Root
	Wu Yao	*Lindera aggregata* (Sims) Kosterm.	Radix linderae	10	Root tuber

**Table 2:** Primer sequences for RT-PCR.

Gene	Type	Sequence
GAPDH	Forward	GGGTCCCAGCTTAGGTTCATCA
	Reverse	GTTCACACCGACCTTCACCATT

Bax	Forward	GTGCAGTGTGTGGCAAAGTCAG
	Reverse	CCAGTTCAGGTGTGGGTGAATG

Bcl-2	Forward	TGAAGCGGTCCGGTGGATA
	Reverse	CAGCATTTGCAGAAGTCCTGTGA

Caspase 3	Forward	AGAGACATTCATGGGCCTGAAATAC
	Reverse	CACCATGGCTTAGAATCACACACAC

**Table 3:** Effect of GQN on tumor growth in H_22_ tumor-bearing mice.

Groups	Dosage(g/kg)	Tumor weight (g)	Inhibitory rate (%)
Model	—	2.30 ± 0.17	—
CTX	0.02	0.95 ± 0.93^*∗∗*^	58.7
GQN (L)	6.92	1.79 ± 0.30^*∗∗##*^	22.17
GQN (M)	13.84	1.42 ± 0.12^*∗∗##*^	38.26
GQN (H)	27.68	1.21 ± 0.10^*∗∗##*^	47.39

^*∗*^ *P* < 0.05 versus model group; ^*∗∗*^*P* < 0.01 versus model group; ^#^*P* < 0.05 versus CTX group; ^##^*P* < 0.01 versus CTX group.

**Table 4:** Effect of GQN on body weight and thymus and spleen indices in mice.

Groups	Dosage (g/kg)	Body weight treated 0 day (g)	Body weight treated 10 days (g)	Thymus index	Spleen index
Model	—	23.68 ± 1.12	35.30 ± 2.40	2.24 ± 0.86	3.52 ± 0.99
CTX	0.02	23.44 ± 2.21	31.48 ± 2.60^*∗*^	0.98 ± 0.25^*∗∗*^	3.05 ± 0.97
GQN (L)	6.92	23.48 ± 1.09	31.59 ± 2.49^*∗*^	1.86 ± 0.60^##^	4.36 ± 1.16^#^
GQN (M)	13.84	22.29 ± 1.25	29.85 ± 1.60^*∗*^	1.60 ± 0.56^##^	4.19 ± 0.91^#^
GQN (H)	27.68	23.57 ± 1.17	29.35 ± 1.89^*∗*^	2.26 ± 0.87^##^	4.49 ± 1.59^##^

^*∗*^ *P* < 0.05 versus model group, ^*∗∗*^*P* < 0.01 versus model group, ^#^*P* < 0.05 versus CTX group, and ^##^*P* < 0.01 versus CTX group.

## Data Availability

The datasets in this paper are available from the corresponding author upon reasonable request.
